# The Potential Role of Probiotics, Especially Butyrate Producers, in the Management of Gastrointestinal Mucositis Induced by Oncologic Chemo-Radiotherapy

**DOI:** 10.3390/ijms25042306

**Published:** 2024-02-15

**Authors:** Massimiliano Cazzaniga, Marco Cardinali, Francesco Di Pierro, Giordano Bruno Zonzini, Chiara Maria Palazzi, Aurora Gregoretti, Nicola Zerbinati, Luigina Guasti, Alexander Bertuccioli

**Affiliations:** 1 Scientific & Research Department, Velleja Research, 20125 Milano, Italyf.dipierro@vellejaresearch.com (F.D.P.); 2 Microbiota International Clinical Society, 10123 Torino, Italyalexander.bertuccioli@uniurb.it (A.B.); 3 Department of Internal Medicine, Infermi Hospital, AUSL Romagna, 47921 Rimini, Italy; 4 Department of Biomolecular Sciences, University of Urbino Carlo Bo, 61122 Urbino, Italy; 5 Department of Medicine and Surgery, University of Insurbia, 21100 Varese, Italyluigina.guasti@uninsubria.it (L.G.)

**Keywords:** probiotics, butyrate, mucositis, radiotherapy, chemotherapy

## Abstract

Many clinical studies have now highlighted how the composition of the intestinal microbiota can regulate the effects of many oncological therapies. In particular, the modulation of microbial composition has been shown to enhance their efficacy and reduce potential side effects. Numerous adverse events induced by chemotherapy and radiotherapy appear to be strongly associated with an alteration in the intestinal microbiota caused by these treatments. This supports the hypothesis that the modulation or correction of the microbiota may decrease the toxic impact of therapies, improving patient compliance and quality of life. Among the most debilitating disorders related to oncological treatments is certainly mucositis, and recent clinical data highlight how the deficiency of short-chain fatty acids, especially butyrate, and specifically the lack of certain bacterial groups responsible for its production (butyrate producers), is strongly associated with this disorder. It is hypothesized that restoring these elements may influence the onset and severity of adverse events. Therefore, the intake of probiotics, especially butyrate producers, and specifically *Clostridium butyricum* (CBM588), currently the only cultivable and usable strain with a history of data proving its safety, could be a valuable ally in oncological therapies, reducing the associated discomfort and improving compliance, efficacy, and quality of life for patients.

## 1. Introduction

**Figure 1:**
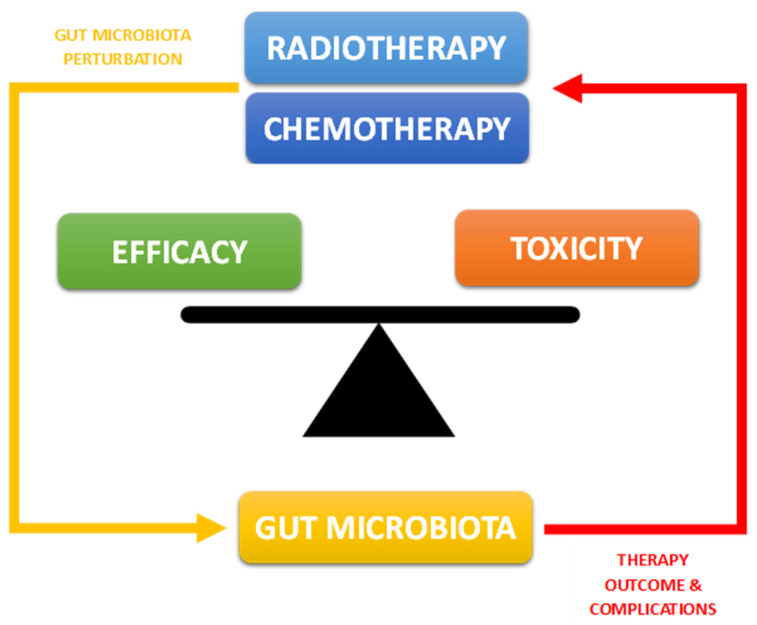
Correlation between intestinal microbiota and oncological chemo-radiotherapy.

In recent decades, the effectiveness of oncological therapies has continually improved, enhancing the prognosis of many cancers. However, despite these advancements, a significant proportion of patients still do not respond adequately to oncological treatments. In this regard, the causes can be attributed to numerous factors, but many studies have now confirmed that the composition of the intestinal microbiota (GM) plays a crucial role in regulating the efficacy of oncological treatments. Our ability to modulate its composition can impact both the treatment’s effectiveness and the occurrence of side effects [1]. In the case of oncological chemotherapy and radiotherapy, a reciprocal influence between the treatment and the GM is widely accepted. The microbiota significantly contributes to the pharmacokinetics and pharmacodynamics of the administered compound (either orally or parenterally) by metabolizing it directly or indirectly through enzyme production. On the other hand, the therapy can also modify/alter the microbial composition, creating conditions for the onset of new concurrent pathologies, as well as indirectly influencing the effectiveness of the treatment itself [2] (Figure 1). One area where scientific results seem to be consolidated and of significant clinical impact is the relationship between the GM and the occurrence of specific side effects, affecting patients’ quality of life and prognosis. This opens up the possibility of using the GM as a new therapeutic target to influence the onset and severity of these issues and ultimately impact the disease prognosis. An example of this is the frequent occurrence of gastrointestinal symptoms, particularly mucositis, during oncological chemo-radiotherapy and how its relationship with a specific microbial profile allows for new control strategies [3,4,5].

## 2. Alterations in the Intestinal Microbiota during Chemo-Radiotherapy

The effect of oncological treatments such as chemotherapy and radiotherapy leads to a series of significant modifications to the gut microbiota (GM), which are now widely documented and subsequently impact the clinical parameters of the treatments themselves. Numerous clinical studies now confirm this influence. One of the most recent and authoritative works on the subject is a systematic review from 2021 [6], which focuses on the effect of radiotherapy on the GM of nearly 3000 patients from 11 clinical studies. The results unequivocally highlight how ionizing radiation profoundly alters the microbial composition, leading to dysbiosis in 81% of patients. Specifically, this involves a decrease in microbial richness and biodiversity linked to an increase in pathogenic bacterial populations, primarily Proteobacteria and Fusobacteria, and a corresponding decrease in beneficial ones such as Faecalibacterium and Bifidobacterium.

Data regarding the impact of chemotherapy on the microbiota are not dissimilar (Table 1) [7]. In a highly significant study [8], for example, conducted, on pediatric patients with acute myeloid leukemia, chemotherapy demonstrated a potent impact on microbial composition, both in terms of richness (estimated more than 100 times lower compared to healthy controls) and in qualitative terms. This was evidenced by a more than 10,000-fold decrease in anaerobic bacteria, compensated by a 100-fold increase in pathogenic bacteria such as Enterococci. The end result was deterioration of the microbial profile in both quantitative and qualitative terms.

In particular, in all studies present in the literature, it is now evident that one of the most frequent and significantly altered microbial characteristics during oncological treatments is the substantial decrease in all bacterial phyla responsible for the production of short-chain fatty acids. This makes these compounds directly associated with side effects and potential targets for complementary therapies to oncological treatments. Given the numerous metabolic roles played by short-chain fatty acids, it is crucial to analyze the implications that may arise from their reduction. Further details and implications of SCFA producer alterations will be thoroughly discussed in Section 3.

## 3. Short-Chain Fatty Acids (SCFAs)

**Figure 2:**
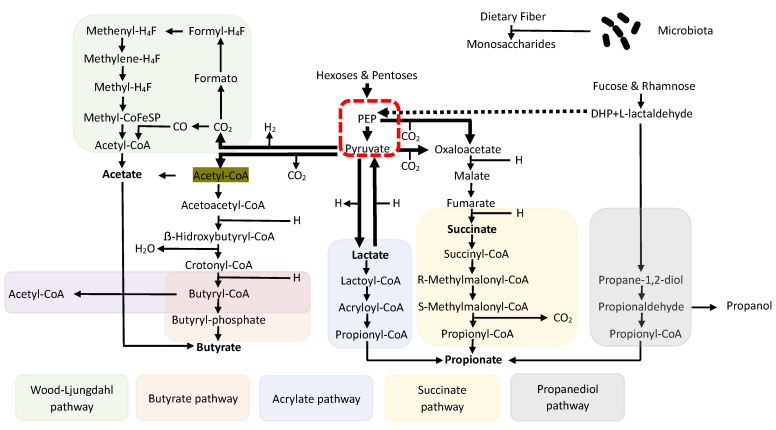
Biosynthesis pathways of SCFAs with fermentation of non-digestible carbohydrates with a common denominator in PEP and cross-feeding phenomena.

Short-Chain Fatty Acids (SCFAs) are, by definition, fatty acids with fewer than six carbon atoms. They are produced by the intestinal microbiota thanks to the ability of certain bacterial groups to ferment specific undigested and absorbed nutrients in the small intestine. In detail, some non-digestible carbohydrates (polysaccharides, oligosaccharides, fibers, inulin, etc.) are degraded into monosaccharide residues through the enzymatic action of bacterial groups, often forming consortia to enhance fermentative capacity (a phenomenon known as cross-feeding). These residues are then catabolized to form a compound called Phospho-enol-pyruvate (PEP), a precursor to pyruvate and a crucial element from which SCFAs, particularly the three most important ones—acetate, propionate, and butyrate—derive through a series of complicated biochemical events [17] (Figure 2).

**Figure 3:**
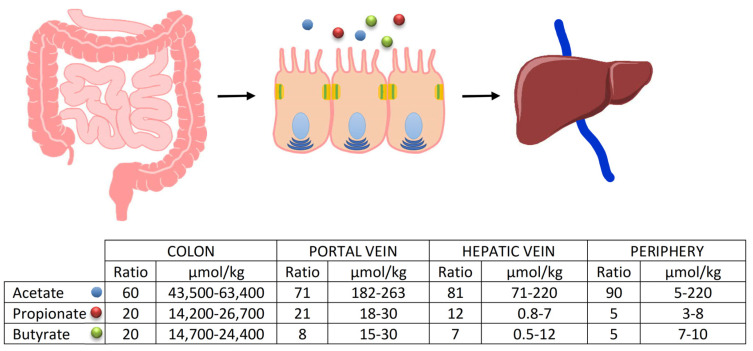
Fate of the SCFAs after their formation. The percentages of passage to the peripheral circulation are evident, as is the important amount of butyrate that remains available to the enterocytes.

Among the bacterial groups suitable for this function, particularly in generating butyrate (the short-chain fatty acid extensively discussed in this work), the most important are undoubtedly the Firmicutes phylum, especially the genera Lachnospiraceae and Ruminococcaceae. These bacteria can produce enzymes such as Butyrate Kinase and Butyrate CoA transferase, responsible for generating the majority of the compound [18]. It is important to note that the quantity of these compounds is not produced equally throughout the intestinal tract but varies significantly depending on the considered segment. It is abundantly represented in the proximal colon, decreasing proportionally as one moves from the distal colon toward the rectum. This is because the vast majority of undigested compounds in the small intestine will be found immediately after the ileocecal valve, where the majority of fermentative action occurs in the proximal colon, thus being the region where bacterial phyla capable of fermentation are most represented [19]. After their formation, the fate of SCFAs, especially the three most important ones, is not the same. Butyrate, for instance, is the main energy source for enterocytes, and it is not surprising that a significant portion (about 70%) of the absorbed amount remains at the level of colon cells, with only a fraction passing into the periphery. The destiny of propionate is different; after colonic absorption, it passes in large quantities through the portal vein to the liver, where it exerts most of its functions, while acetate (the most abundantly produced fatty acid) almost entirely passes through the liver, and then, into the peripheral circulation [20] (Figure 3). However, even though it is clear that only a small amount of propionate, and especially butyrate, reaches the periphery, and thus, the organs, they have numerous functions, and their absence or low production can lead to various issues. This is particularly relevant during oncological treatments, particularly in those areas of the colon already characterized by reduced production of basic SCFA due to a reduction in the bacterial phyla that produce them [7,8,9,10,11,12,13,14,15,16] (Table 1). The most protected areas, consequently, will be those of the proximal colon where the high production of SCFA leads to better defense.

## 4. Oncological-Treatment-Induced Mucositis and Its Correlation with SCFAs

Mucositis is, by definition, a variable inflammatory condition in terms of degree and severity affecting the gastrointestinal (and oropharyngeal) mucosa in oncology patients undergoing chemotherapy and radiotherapy. It is an extremely debilitating complication characterized by inflammation and loss of the epithelial lining of the intestinal barrier [21]. Symptoms vary widely and can be intense, primarily manifesting as crampy abdominal pain, nausea, loss of appetite, weight loss, and profuse watery diarrhea. It is estimated that more than half of patients undergoing oncological treatments experience mucositis, and the frequency of this phenomenon increases when chemotherapy and radiotherapy are administered simultaneously to the same patient, amplifying the occurrence by about 5 times [22,23]. This implies, in addition to increased costs and hospitalizations, reduced compliance in terms of both the execution and dosage of oncological treatments, thereby impacting the prognosis of diseases. In 2004, Sonis et al. [24] described a histopathological model divided into five phases or sequences to explain the onset, progression, and healing of the event.

**Figure 4:**
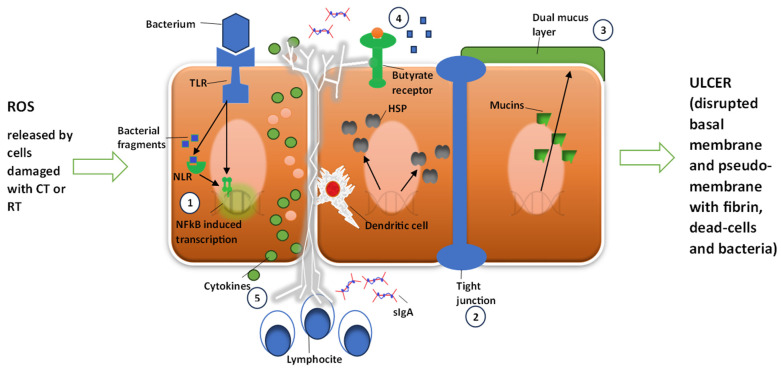
Sonis model of mucositis pathogenesis and bacterial contribution to cell damage amplification and degree. Cells damaged by chemotherapy (CT) or radiotherapy (RT) release radical oxygen species (ROS). Damage is further amplified by NF-κB activation by ROS. Bacteria could 1—contribute to NF-κB activation through bacterial fragments’ activation of NLR and through the TLR receptor pathway; 2—increase intestinal permeability; 3—influence mucus layer composition; 4—reduce resistance to harmful stimuli and reduce epithelial repair; and 5—contribute to activation and release of immune effector molecules. HSP: heat shock protein; sIgA: serum immunoglobulin A; TLR: Toll-like receptor; NLR: nucleotide-binding domain leucine-rich repeat-containing proteins.

The five phases were essentially defined as follows: Phase one is Initiation, characterized by an inflammatory phase of the mucosa induced by chemotherapeutic agents and/or ionizing radiation. It particularly recognized the formation of reactive oxygen species (ROS) following oxidative stress. Phases 2–3 comprise a dual phase of initial damage and amplification of the inflammatory component characterized by cellular DNA damage and the activation of NF-κB and TNF. Phase 4 is a phase defined as Ulceration, extremely important as it results from the destruction of the basement membrane and an increase in barrier permeability (tight junctions) with a decrease in protective properties. According to this model, in this phase, the passage of pathogenic bacteria through the barrier occurs. Finally, Phase 5 is a phase defined as Healing, where normal wall functionality is restored following the cessation of the chemo-radiotherapeutic stimulus. Recently, in light of recent discoveries about the microbiota and its functions, this model has been substantially adapted. In contrast to the original model, the importance of the bacterial population is not only seen as a consequence of the described inflammatory and ulcerative process but also, and above all, as its cause [3]. This new evidence should also be taken into consideration when reconsidering and deepening current knowledge on different dietary models, including the Mediterranean diet [25], considering not only the nutritional aspects but also, more broadly, the effects that a specific dietary pattern can have at an “ecosystemic” level, including the intestinal microbiota and everything that can cascade from it. According to the new evidence, oncological treatment alters the intestinal bacterial composition, and the newly created population is the basis of the inflammatory and membrane alteration phenomena typical of mucositis (Figure 4) (see Figure 1 and Figure 2).

**Figure 5:**
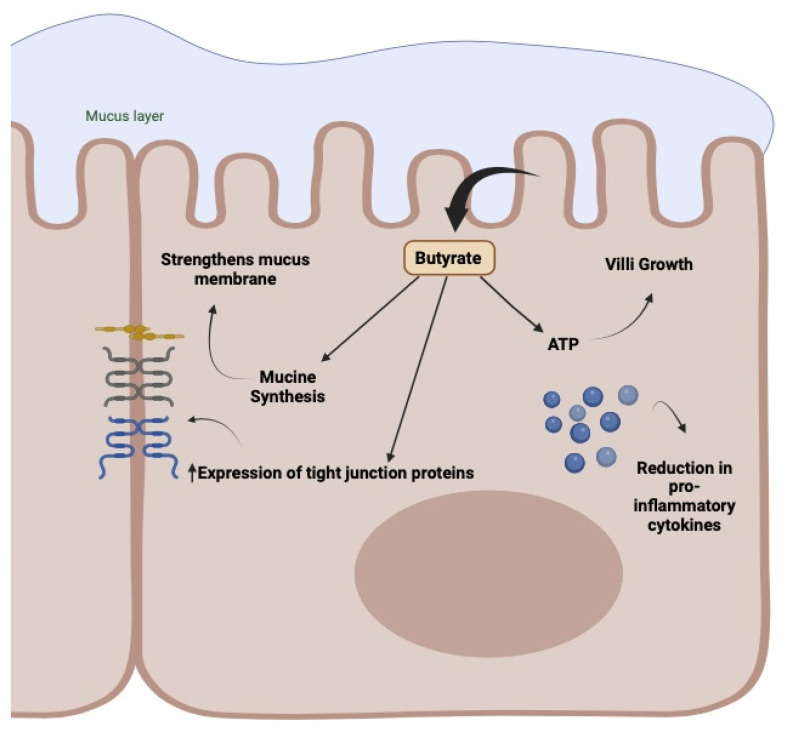
Activity of butyrate in the enterocyte membrane. Mucus production, wall protection with compaction of tight junctions, energy production, and inflammatory and immune regulation.

In practice, as observed earlier, oncological treatments generate a modification of the bacterial population, essentially characterized by a decrease in the Gram+ component, especially that associated with Firmicutes and butyrate producers, with a consequent increase in Gram- bacteria, particularly Fusobacterium and Proteobacteria carrying LPS. The presence of higher amounts of LPS is responsible for the onset of inflammatory pathways, mainly due to their ability to bind and activate TLR4. Moreover, the simultaneous disappearance of/reduction in butyrate-producing bacteria will result in the reduced production of protective mucus, solidity of tight junctions, increased wall permeability, and altered maturation of the immune component in the lamina propria with a decrease in Treg and an increase in Th17, as all these parameters are directly connected to the quantity and activity of butyrate [26,27,28] (Figure 5).

The use of probiotics appears to be a promising therapy in the management of complications induced by chemotherapy treatments. For example, bifidobacterial and lactobacilli can increase the expression of tight junction proteins and consequently restore the intestinal barrier function compromised by chemotherapy treatment. The use of SCFAs also leads to the strengthening of tight junctions, contributing to the maintenance of a healthy intestinal barrier. The use of yeasts such as Saccharomyces cerevisiae has also been shown to prevent the loss of goblet cells, preserve the architecture of the intestinal mucosa, and reduce mucosal inflammation [29]. Furthermore, the oral administration of *Clostridium butyricum* has been shown to increase and improve overall survival in patients with non-small-cell lung cancer treated with chemoimmunotherapy thanks to the production of butyric acid which, by improving the host’s immunity and promoting the growth of beneficial populations such as Bifidobacterium, guarantees a better therapeutic outcome [30].

## 5. The Increase in Circulating Butyrate and Its Impact on the Treatment of Gastrointestinal Disorders

What is highlighted makes it clear that our ability to increase the level of SCFAs, especially butyrate, in our body could significantly help in controlling gastrointestinal disorders, including those related to oncological treatments. As evident from the described formation mechanism, there are essentially two ways to increase the level of circulating butyric acid, both aimed at enhancing its endogenous production. The first and more understandable approach is to stimulate greater production of the compound by providing a higher amount of material to be fermented to the bacteria responsible for this process. This action can be achieved through a specific type of diet, particularly one rich in prebiotics. It is logical to deduce that increasing the amount of indigestible material in the small intestine, such as consuming a fiber-rich diet, will provide adequate quantities of oligo- and polysaccharides to the microbiota, effectively promoting the increased production of SCFAs and butyrate. However, the modulation of the SCFA profile is strongly influenced by the type of fiber introduced in the diet and by the structure and dose consumed. In general, a higher dose of fiber in the diet has a greater influence on the microbial ecosystem than lower doses: the consumption of a diet rich in whole grains (32 g/day of total fiber) led to an increase in SCFAs, different from a diet low in dietary fiber [31].

A recent review [31] evaluated the impact of different types of fibers on the production of SCFA. Arabinoxylan oligosaccharides (AXOS) were considered, for which higher levels were evaluated compared to baseline following their administration (2.2 g/day for 30 days); the use of lupine fiber, at a dose of 25 g/day, also led to a significant increase in SCFAs compared to baseline. Furthermore, different types of resistant starch type 4 (RS4) resulted in different effects on the synthesis of SCFAs: corn and tapioca RS4 led to increased values of butyrate and propionate, respectively, determined based on the enrichment of *E. rectale* and *P. distasonis*, following a dose of 35 g/day. The consumption of 5 g/day of xylo-oligosaccharides (XOS) or 3 g/day of inulin together with 1 g/day of XOS for a period of 4 weeks also led to an increase in total levels of SCFAs. XOS has also promoted an increase in strains such as *Akkermansia* and *Faecalibacterium*. Numerous studies now confirm the effectiveness of this approach [31].

However, what is evident is that this type of intervention may be less efficient if the issue is supported by a modification of the microbiota, particularly a reduction in the bacterial phyla responsible for SCFA formation. In essence, it becomes clear that increasing the “food” for butyrate-producing bacteria is quite pointless when the presence of these bacteria has drastically decreased due to oncological treatments.

It is likely that the second approach to the problem, represented by our ability to modulate the microbiota by increasing the presence of butyrate-producing bacterial phyla, may be more efficient. Recent studies have already highlighted the possibility of managing complications related to oncological therapies using this intervention model. One of the most authoritative studies has recently demonstrated how the intake of a combination of probiotics can reduce such complications, especially diarrhea, in patients undergoing chemotherapy for colorectal cancer [32].

All of this revolves around our ability to cultivate, and then, consume probiotics suitable for this purpose—bacterial groups that, when introduced into our body, can colonize and produce adequate amounts of SCFAs. Of course, theory differs significantly from practice, and our ability to cultivate such probiotics on a large scale is currently very limited. Most bacteria suitable for this purpose are quite fragile, making their production very challenging, if not impossible. However, there are exceptions, and the main one is represented by a bacterial genus, “*Clostridium butyricum*”, which appears to be suitable for this purpose.

## 6. *Clostridium butyricum* CBM 588

*Clostridium butyricum* (CB) is a well-known beneficial symbiotic, Gram-positive butyrate producer and obligate anaerobic spore former present in various environments, especially in the soil. It is found in approximately 20% of adults [33] and is naturally present in the colon, where it ferments non-digestible carbohydrates, producing butyric acid. Scientific data regarding this bacterium are extensive and noteworthy, as it is already widely used in some Eastern countries (Japan, Korea, and China) as an effective and safe remedy for various gastrointestinal symptoms, particularly intractable diarrhea and antibiotic-induced colitis [34]. *Clostridium butyricum* CBM 588 (CBM 588) has demonstrated all the beneficial characteristics typical of a butyrate producer, such as mucin production for the protection of the intestinal wall, the reinforcement of tight junctions (essential epithelial features in the onset of diarrheal phenomena), as well as the regulation of inflammatory and immune components, [35]. Currently, it is the only cultivable and usable strain with a history of data proving its safety.

**Figure 6:**
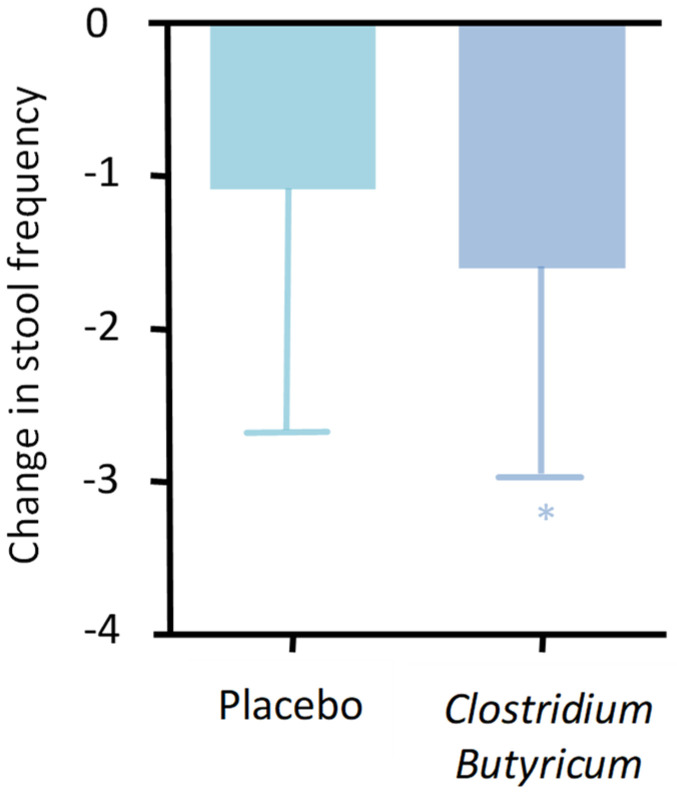
Change in bowel movement frequency between treatment and placebo (*p* = 0.035). * *p* < 0.05.

The clinical effects related to these capabilities are now recognized and significant. CBM 558’s administration appears suitable in controlling gastrointestinal infections, especially, but not exclusively, antibiotic-induced ones. Clinical results are now evident concerning its ability to reduce infections from important pathogens such as *Escherichia Coli*, *Helicobacter pylori*, *Staphylococcus aureus*, and *Salmonella* spp. [36]. Equally evident are the data regarding its ability to control diarrhea. There are ample data in this regard, and one of the most suitable studies confirming this aspect is a 2017 double-blind, placebo-controlled randomized controlled trial (RCT) conducted on patients suffering from inflammatory bowel disease (IBD) [37]. The study aimed to assess the efficacy and safety of CB in controlling diarrhea in patients with IBD. Two hundred patients who took a placebo or CB for 4 weeks were evaluated, highlighting a clear and statistically significant improvement in symptoms related to the IBD condition in the treatment group. There was also an improvement in the quality of life of the patients, and notably, a drastic reduction in diarrheal events (Figure 6).

As analyzed in a previous study of ours, CBM588 also exhibits additional mechanisms of action that deserve further exploration [38].

### 6.1. Clostridium butyricum CBM 588’s Effects in Animal Models

Hagihara et al. have indicated that the use of CBM588 is associated with increased production of IL-17 by intraepithelial γδ T lymphocytes, acting as the first line of defense in the colon lamina propria [39]. In this context, IL-17 contributes to maintaining the integrity of the intestinal barrier by enhancing the production of structural proteins of tight junctions [40]. This exerts a protective effect and promotes repair processes. Furthermore, the modulating effects on inflammatory processes due to the increased production of lipid metabolites with anti-inflammatory properties, such as palmitoleic acids, prostaglandin metabolites, and pro-resolutive mediators, are also described for CBM588 in the murine model. These mediators include Protectin D1, which contributes to stimulating the secretion of IL-10 by colon T cells [39,41,42], thus favoring mucosal integrity.

Zhao et al. reported that the administration of *C. butyricum* is associated with a reduction in visceral intestinal hypersensitivity and mucosal inflammation [43]. Analyzing the effects related to the use of CBM588 in three different gastric ulcer models (induced by alcohol, stress, and pyloric ligation), reduced mucosal damage and an improvement in the inflammatory state were observed, resulting in a reduction in oxidative stress [44].

As indicated by Stoeva et al. in experimental studies, the CBM588 strain has proven to be effective in controlling Clostridium difficile infection and managing dyslipidemia, glycemic dysregulation, and intestinal inflammation caused by dextran sulfate inoculation, thanks to its ability to produce butyrate. The authors have also described how other strains of *Clostridium butyricum* have shown clinical action in IBS-D, again due to butyrate production [45].

Human studies demonstrate that the use of CBM588 is associated with an increase in Bifidobacterium and Lactobacillus, a decrease in Enterococcus and Enterobacteriaceae, and the restoration of fecal anaerobes after antibiotic treatment [35,46,47], without altering the overall diversity of the intestinal microbiota.

### 6.2. Clostridium butyricum CBM 588’s Effects in Clinical Evaluations

As previously mentioned, CBM588 has demonstrated a positive safety profile, good colonization capabilities, and the ability to promote a eubiotic intestinal environment through its production of butyric acid.

Seki et al. have described how the concurrent use of CBM588 in children with upper respiratory tract infections or gastroenteritis treated with antibiotics is associated with a significant reduction in episodes of diarrhea and a decrease in anaerobic bacteria and bifidobacteria. In subjects treated only with antibiotics, diarrhea occurred in 59% of cases, while in subjects who received CBM588 halfway through antibiotic treatment, it occurred in 5% of cases, and in those who took it from the beginning, it occurred in 9% of cases [34]. These results suggest the utility of CBM588 in preventing antibiotic-induced diarrhea in children.

Imase et al. reported that in the context of Helicobacter pylori eradication therapy, the use of CBM588 is associated with an incidence of 14% for diarrhea or loose stools, compared to 43% in subjects who exclusively followed antibiotic therapy. Doubling the dose of CBM588, none of the analyzed subjects reported diarrhea or loose stools [46].

Yasueda et al., examining the onset of pouchitis in subjects undergoing total proctocolectomy with anal anastomosis of the ileal pouch (IPAA), observed that the use of CBM588 was associated with only one episode out of nine observed subjects, compared to four subjects out of eight who took a placebo and developed pouchitis [48].

### 6.3. Clostridium butyricum CBM 588’s Effects on Digestive Disease Models

Examining the potential functional impacts exerted by CBM588, several authors have provided interesting results in the context of treating digestive tract disorders. The studied applications include irritable bowel syndrome (IBS), ulcerative colitis (UC), gastric ulcer, and intestinal dysbiosis. 

These data can represent an intriguing analytical model for common pathophysiological aspects or those that nonetheless exhibit significant elements in the previously discussed oncological scenarios, providing valuable insights into understanding CBM 588’s possible applications.

Araki et al. illustrated, in a murine model, how the administration of CMB588 is associated with a reduction in diarrhea and mucosal damage induced by dextran sulfate sodium (DSS) [49]. Similarly, Hayashi et al. highlighted how the administration of CBM588 prevents experimentally induced colitis in a murine model [50]. Furthermore, as reported by Okamoto et al., the administration of CBM588 initiated one week before colitis induction in an experimental model, with DSS, is correlated with a significantly lower ulceration index and myeloperoxidase (MPO) activity. At the same time, there is an observed increase in proliferating cells (PCNA) around lesions, along with simultaneous higher levels of Lactobacillus and Eubacterium, as well as increased levels of butyrate, propionate, and acetate at the cecal level [51].

In an experimentally induced IBS murine model, Zhao et al. described conceptually similar results, reporting a reduction in intestinal visceral hypersensitivity and mucosal inflammation associated with the administration of *C. butyricum* [43].

Analyzing the effects of CBM588 administration in three different models of gastric ulcer (alcohol-induced, stress-induced, and pyloric ligation), a reduction in mucosal damage and an improvement in the inflammatory state were observed, accompanied by a decrease in oxidative stress, consistent with previously described mechanisms [44].

Consistent with previous results, studies conducted on human models indicate that CBM588 administration is associated with an increase in Bifidobacterium and Lactobacillus, a decrease in Enterococcus and Enterobacteriaceae, and the recovery of fecal anaerobes following antibiotic treatment, all without altering the overall diversity of the intestinal microbiota [47].

This, in a general context, involves an increase in Firmicutes, among which we find some of the main producers of SCFAs, and a decrease in Bacteroidetes, thus also limiting the presence of LPS with an overall less inflammatory profile.

The effects described by the study of these models establish an intriguing interplay between physiological effects and effects on the intestinal microbiota. Synergistically, these factors may contribute to the management of the pathology, with functional effects potentially beneficial even in the context of the discussed oncological conditions.

The combination of these results suggests a consideration regarding the effectiveness demonstrated in the examined studies on clinical parameters such as colitis, diarrhea, etc. Taken together, these findings appear to strengthen the hypothesis of using CBM588 to modulate the overlapping aspects discussed, especially when induced by oncological therapies. From this perspective, it is crucial to recall the potential actions both at the physiological level and within the microbiotic ecosystem.

## 7. The Potential Oncological Applications of *Clostridium butyricum* CBM 588

Therefore, considering all these data, it is plausible that CB and the butyrate it produces could prove to be dramatically effective as a complementary treatment to oncological therapies (especially radiotherapy and chemotherapy). Assuming the established relationship between the microbiota and oncological therapies, especially the impact of dysbiosis on their efficacy and toxicity, it becomes intuitive that our ability to modulate the microbiota before or during the therapeutic intervention can indirectly impact the outcome of the disease, promoting treatment compliance and regulating dosage and toxicity. Controlling phenomena such as mucositis will undoubtedly enhance the effectiveness of oncological chemo-radiotherapy, allowing patients to better tolerate the treatment without having to modify or even suspend the dosage. The compound’s ability to regulate biological aspects such as the protection of the enterocyte membrane and the modulation of inflammatory and immune pathways will inevitably impact the reduction in side effects and, consequently, the course of the disease.

There are many areas of application for this treatment. Firstly, its efficacy is likely to be evident when used preventively (before the start of treatment) as well as during therapy (when symptoms appear). Preventive use is justified by studies demonstrating that the analysis of the intestinal microbiota can be a predictive factor for the onset of diarrheal symptoms. For example, a study published in 2015 [38] showed that the presence of specific microbial conditions before the start of radiotherapy (such as an altered Firmicutes/Bacteroidetes ratio) can be used to predict patients who will develop diarrheal symptoms, making it intuitive that the preventive correction of this situation could reduce its incidence and, consequently, enhance treatment efficacy. Regardless of the intervention phase, there are many scenarios in which such a treatment could be successful. In practice, it could be used in all patients undergoing or scheduled for radiotherapy (head–neck, thoracic, pelvic, or gastrointestinal) and/or chemotherapy, especially in those pharmacological regimens burdened by known and disabling gastrointestinal disturbances. Some compelling data in the literature seem to suggest the use of CB treatment not only in chemo-radiotherapeutic regimens but also during oncological therapies of different natures [30]. For instance, it could be employed during the use of CDK4/6 inhibitors in hormone-responsive breast cancer, which is often associated with diarrheal symptoms. Additionally, during immunotherapeutic treatments, the ability of SCFAs, especially butyrate, to enhance the effectiveness of CPIs (checkpoint inhibitors) is now evident [52]. 

## 8. Limitations and Future Developments

Many of the works taken into consideration are of a different nature, such as clinical studies, reviews, or meta-analyses. This obviously leads to a lack of uniformity from the point of view of the executive criteria and from the point of view of the overall number of evaluations carried out, especially for some applications, constituting a potential limitation of our analysis. But the coherence of the picture that emerges from their examination suggests the validity of the hypothesis made. These data, in light of the very high safety profile of CBM588 demonstrated by the fact that only three cases of bacteremia in clinically unstable patients are reported in the literature [53], and based on the successful use of CBM588 by other authors in critically ill patients [54], against much more numerous cases found for Lactobacilli and Bifidobacteria [55], could suggest the need to plan and carry out clinical evaluations that can provide us with even more uniform and certainly more numerous data for the treatments taken into consideration.

## 9. Conclusions

Unfortunately, standard treatments, to date, have not completely resolved the significant issues caused by the onset of mucositis. This, as discussed, not only has a significant impact on quality of life but also has important clinical repercussions. The hindrance to normal food dynamics leads to more rapid and impactful deterioration of the patient’s overall condition. From this perspective, the search for new solutions can be considered a true contribution to clinical practice. In the treatment of chemotherapy-induced diarrhea, which involves approximately 50–80% of patients, the most used active ingredients are loperamide, a peripherally limited mu opioid receptor agonist that can inhibit enteric neurons and reduce intestinal motility and octreotide, a synthetic analogue of somatostatin. However, prolonged use of the latter can cause deleterious effects on the cardiovascular system, central nervous system, and endocrine system [56]. The use of butyric acid could be an important tool in addition to those conventionally employed. In particular, the opportunity to produce it directly at the mucosal level through the administration of *Clostridium butyricum* CBM 588 could be, in addition to an innovative form of delivery, a potential way to attempt to reduce the patient’s dependence on the product for the duration of the microorganism’s presence in the intestinal environment.

Furthermore, the documented protective properties of *Clostridium butyricum* CBM 588 against pathogenic microorganisms allow for an additional benefit regardless of butyrate production. The clinical validation of this potential approach is a critically important element, considering the high degree of complexity of the issue. Planning an appropriate study is a significantly challenging task but one that must be addressed as soon as possible to evaluate the correctness of this potential application.

In conclusion, the administration of butyric acid and its endogenous production by *Clostridium butyricum* CBM 588 could constitute a new potential application strategy capable of integrating with the treatments already in use for the control and prevention of mucositis in oncology patients, potentially contributing to clinical outcomes and quality of life.

According to the European Commission, *Clostridium butyricum* (CBM588) meets the safety criteria established by Article 3 of Regulation (EC) n. 258/97 of the European Parliament and of the Council concerning new products and new food ingredients. However, its supplementation requires further investigation to provide a more complete understanding of the mechanisms involved in its beneficial potential and evaluate its effectiveness in different contexts and conditions.

## Figures and Tables

**Table 1:** Influence of chemotherapeutic treatments on intestinal microbiota profiles [7].

Chemotherapeutic Treatment	Microbiota Modifications	Refs
5-Fluorouracil	Increase in Gram-negative anaerobes Increased translocation to mesenteric lymph nodes Increase in *Clostridium* spp., *Staphylococcus* spp., and *Escherichia coli* and decrease in *Lactobacillus* spp. and *Bacteroides* spp.	[9,10]
Cycles I and Il: high-dose cytarabine, daunorubicin, and etoposide; cycle Ill: amsacrine, high-dose cytarabine, and etoposide; cycle IV: mitoxantrone and high-dose cytarabine [9]	Lower total number and diversity of intestinal bacteria; decrease in *Bacteroides* spp., Clostridium cluster XIVa, Faecalibacterium prausnitzii, and *Bifidobacterium* spp.; increase in pathogenic enterococci and decrease in streptococci	[8]
Cyclophosphamide	Decrease in Clostridium cluster XIa, *Roseburia*, *Lachnospiraceae*, *Coprococcus*, *lactobacilli*, and *enterococci* Increased translocation of Gram-positive species to mesenteric lymph nodes and spleen Increased *Escherichia coli*, *Pseudomonas*, *Enterobacteriaceae*, and *enterococci* Increased Firmicutes/Bacteroidetes ratio Increased Actinobacteria, Bacteroidia, Alphaproteobacteria, *Lachnospiraceae*, *Coriobacteriaceae*, *Lactobacillaceae*, and *Staphylococcaceae*; decreased Bacteroidetes, *Bacilli*, *Clostridia*, *Coriobacteria*, *Mollicutes*, *Prevotellaceae*, 524-7, Alcaligenaceae, and Rhodospinillacene; disappeared Verrucomicrobia and Streptococcacede	[11,12,13]
Irinotecan	Increased Clostridium cluster XI (including *Peptoclostridium difficile*) and Enterobacteriaceae	[14]
High-dose carmustine, etoposide, aracytine, and melphalan	Increased Proteobacteria, decreased Firmicutes and Actinobacteria	[15]
Gemcitabine	Increased Proteobacteria, Verrucomicrobia, Akkermansia muciniphila, *Escherichia coli*, and *Peptoclostridium difficile* Decreased Firmicutes, *Bacteroidetes*, *Bacteroidales*, *Lachnospiraceae*, *Ruminococcaceae*, *Bacteroides acidifaciens*, and *Lactobacillus animalis*	[16]

## Data Availability

Data are contained within the article.
